# Meditation in an academic medical institution to improve student mental health: a pilot study

**DOI:** 10.3389/fdgth.2026.1957495

**Published:** 2026-10-12

**Authors:** Marie Vazquez Morgan, Suzanne Tinsley

**Affiliations:** Department of Student Mental Health, Advocacy and Wellness, Louisiana State University Health Shreveport, Shreveport, LA, United States

**Keywords:** anxiety, meditation, GAD-7, graduate students, institutional wellness, virtual reality

## Abstract

**Introduction:**

: Anxiety is common among graduate and professional students. Meditation has been shown to reduce anxiety symptoms, and virtual reality (VR)-guided meditation has emerged as a more immersive approach that may increase engagement. This pilot study evaluated the feasibility, adherence, and preliminary anxiety outcomes associated with VR-guided and audio-guided meditation among health professions students.

**Methods:**

Feasibility was assessed using recruitment, enrollment, retention, assessment completion, intervention delivery, and attendance-data capture. Adherence was defined as the number of QR-code-recorded meditation-session attendances during the 8-week intervention. Anxiety outcomes were assessed using the GAD-7. Within-group changes were examined using paired t tests and Wilcoxon signed-rank tests, and between-group differences in change scores were explored with an independent-samples t test. Spearman correlation analysis examined the association between attendance and reduction in anxiety.

**Results:**

Thirty-five students were assessed for eligibility, 22 were randomized, and all completed the intervention and post-intervention assessment. Both modalities were delivered as planned, and 226 session attendances were recorded. Participants in the VR condition completed a mean of 12.00 recorded sessions (SD = 5.42; range, 1–17), corresponding to 75.0% of the prescribed minimum, whereas participants in the audio-guided condition completed a mean of 8.55 sessions (SD = 5.22; range, 1–18), corresponding to 53.4% of the prescribed minimum. GAD-7 scores decreased significantly within both groups. A linear mixed-effects model demonstrated a significant main effect of time (*z* = 6.80, *p* < .001) but no significant Condition×Time interaction (*z* = −0.28, *p* = .783). Consistent with this finding, the difference in change scores between groups was not significant, t(20) = 0.27, *p* = .786, Cohen's d = 0.12. Recorded attendance was not associated with improvement in GAD-7 scores.

**Conclusion:**

Both meditation modalities were successfully implemented, and all participants completed the study. Recorded attendance was greater in the VR condition; however, adherence varied substantially, and attendance was measured through check-in records only. Anxiety symptoms decreased in both groups, but the absence of a control group and small pilot sample preclude conclusions regarding efficacy or equivalence.

## Introduction

Student mental health is an increasing concern, particularly in graduate and professional programs where students are exposed to intense coursework, frequent examinations, and rigorous clinical training (1, 2)_._ The COVID 19 pandemic may have increased awareness of student mental health, but anxiety and other psychological disorders were prevalent among college students even prior to the pandemic. The magnitude of the problem may be seen from the data from national counseling centers. The Center for Collegiate Mental Health estimates that around 62.7% of students who sought counseling had anxiety related concerns (3)_._

Students in medical and other health profession programs confront unique challenges. Psychological distress is exacerbated by heavy academic workloads, lack of personal time for self-care, poor sleep, and anxieties about seeking mental health (1, 2)_._ In a meta-analysis Quek et al. pooled data from 69 studies involving 40,348 medical students and found a global prevalence of anxiety of 33.8%, with approximately 1 in 3 medical students worldwide experiencing anxiety (1). Further, only a small percentage of students who screened positive for poor mental health sought professional help. Concerns about stigma, confidentiality, seeming weak and possibly professional consequences still prevent many students from seeking help (4)_,_

The COVID 19 pandemic has increased the stress level for an already stressful educational environment. Students faced disruption to their academic preparation, less social interaction, uncertainty about future education and employment, financial difficulty, worries about illness, and fewer opportunities to rely on existing support structures (4–6)_._ These studies also indicated increased anxiety, and stress among students (4, 6)_._

Because many of the responsibilities associated with graduate and professional education are unavoidable, colleges and universities have placed increased focus on preventive techniques that build resilience and improve students’ ability to handle stress (2, 7)_._ Healthy lifestyle behaviors, such as regular physical activity, sleep, healthy food, social support, counseling and stress management strategies such as meditation, have been associated with greater well-being and quality of life among medical students (2, 7)_._

Meditation is an attractive strategy because it is affordable, flexible, and reasonably easy to include into busy academic schedules (8–10)_._ Meditation enhances awareness of the present moment through controlled breathing, body awareness and guided visualization (11)_._ Meditation has been found to lower anxiety by decreasing rumination, enhancing emotional regulation, and encouraging the body's relaxation response (9–11)_._ Short, guided meditation sessions may be especially practical for students, as they require little training and can be more easily fitted into busy daily schedules than lengthy meditation programs (12)_._

Meditation is more easily employed and accessible thanks to new technology. Some students experience difficulty meditating in situations that include distractions, interruptions, noise or lack of solitude. A possible answer is virtual reality that immerses users in a controlled environment using a helmet mounted display, decreasing distractions from the outside world and enabling prolonged attention in meditation (13–15)_._ High immersion VR is a computer generated 360-degree environment viewed through a helmet mounted display, enhancing the user's sense of immersion and realism (16)_._

Being completely immersed in a virtual environment may amplify the meditative experience. VR may assist students in temporarily detaching from academic concerns and focusing more fully on guided meditation by limiting competing sensory input and providing peaceful natural or virtual environments (17, 18)_._

Virtual reality has been investigated as a means of treatment for mental health disorders, in particular as a form of exposure therapy for phobias, social anxiety, and post-traumatic stress disorder (8)_._ Research into VR meditation and relaxation continues to increase (14, 19). Riches et al. (2023) conducted a systematic review and found VR relaxation therapies to be promising low intensity techniques for lowering stress and improving relaxation, particularly for people with anxiety related illnesses (15). At the same time, the authors stressed the necessity for bigger randomized controlled studies, standardized outcome measurements, longer follow up periods, and direct comparisons with established relaxation techniques.

Promising results have been also obtained in studies with college students (12, 16, 20)_._ Kaplan Rakowski, Johnson and Wojdynski (2021) contrasted VR meditation with video-based meditation prior to an exam and found both useful. Participants utilizing VR demonstrated greater reductions in pre-examination anxiety and improved academic performance than those using video-based meditation (16)_._ The study was unable to evaluate if recurrent VR meditation leads to bigger improvements in generalized anxiety over time, given the intervention was limited to one pre-examination session.

Other research has shown that short VR meditation sessions lead to higher immediate increases in engagement than audio-only meditation, but both are better than no meditation (14, 16)_._ These findings suggest that immersive environments may enhance engagement during meditation; however, current evidence does not consistently demonstrate superior anxiety reduction compared with traditional meditation approaches. For example, Jo et al. found that both VR-guided and imagery-based meditation significantly reduced stress and negative affect, with no significant differences between groups in overall stress-reduction outcomes (21, 23). Audio guided meditation is still an appealing choice as it is inexpensive, easily accessible, and requires no specific equipment.

Louisiana State University Health Sciences Center Shreveport understands the importance of supporting the mental health and well-being of graduate students, medical students and residents. The Office of Institutional Wellness and the dedicated meditation suites are a testament to the institution's commitment to accessible, preventive wellness resources. This initiative draws on prior work by Vazquez Morgan (2021) that described the value of comprehensive wellness programs to promote resilience, encourage self-care, and establish a culture of well-being inside academic health facilities (4)_._ These resources offer a chance to examine guided VR meditation in an actual institutional wellness context. VR guided meditation vs audio guided meditation is especially critical to assess because these modalities are easy to execute and extremely scalable across academic institutions.

This pilot study was designed to evaluate the feasibility, adherence, and preliminary anxiety outcomes associated with virtual reality and audio-guided meditation interventions among health professions students. Given the exploratory nature of the study and limited sample size, the investigation was not intended to provide a definitive evaluation of the comparative efficacy of VR versus audio-guided meditation but rather to inform the design of future adequately powered trials.

## Materials/methods

The objectives of this pilot study were:

1. To evaluate the feasibility of implementing VR-guided and audio-guided meditation interventions among health professions students, as assessed by recruitment, enrollment, retention, completion of study assessments, successful intervention delivery, and capture of meditation-session attendance.
2. To describe participant adherence to each intervention, operationalized as the number of QR-code-recorded meditation-session attendances during the 8-week intervention.
3. To estimate preliminary within-group changes in anxiety symptoms following participation in each intervention and explore differences in GAD-7 change scores between intervention groups.
4. To explore the relationship between meditation-session attendance and changes in anxiety symptoms.

The study was approved by the Louisiana State University Health Sciences Center–Shreveport (LSUHS) Institutional Review Board (IRB Protocol No. 00002460), and all participants provided written informed consent prior to participation.

Inclusion criteria included students over the age of 18, currently enrolled at LSU Health Shreveport in the health professions, whose baseline GAD-7 scores indicated elevated symptoms of generalized anxiety, with scores of 8 or higher suggesting clinically meaningful anxiety. Exclusion criteria included students unable to complete screening or protocol, or those with a history of seizures, or severe eye impairment, which would limit the ability to engage in the VR experience, or history of motion sickness.

Participants were actively recruited through flyers advertising the study. A total of 35 students were assessed for eligibility. Of these, 13 were excluded, including 10 who did not meet the inclusion criteria or were otherwise ineligible due to medical conditions and 3 who declined to participate.

All participants went through the screening process, which included completing the GAD-7. The GAD-7 is a 7-item self-report scale that asks subjects to identify how much they were bothered by each of 7 symptoms during the previous 2 weeks. Response options include, “not at all,” “several days,” “more than half the days,” and “nearly every day,” scored 0, 1, 2, and 3, respectively. Total score ranges from 0 to 21 with higher scores indicating higher anxiety and a higher likelihood of meeting criterion for GAD. A score of 8 has been shown to have a 92% sensitivity and a 76% specificity in relation to a diagnosis of GAD (5). Although a cutoff score of 10 is commonly used to identify probable generalized anxiety disorder, a threshold of 8 was selected for this study to increase screening sensitivity and identify students with clinically meaningful anxiety symptoms who may benefit from early intervention. The GAD-7 has previously demonstrated an internal consistency of 0.92 and test-retest reliability of 0.83 (22)_._

The screening process also included obtaining a medical history and a list of current medications. Those who chose to participate in the study gave informed consent.

Following informed consent, participants were assigned to either the VR-guided meditation group (*n* = 11) or the audio-guided meditation group (*n* = 11) using a computer-generated random allocation sequence. Simple randomization was used without blocking or stratification. Assignment occurred after eligibility screening and informed consent had been completed.

Because this pilot study involved a small sample and was conducted at a single institution, formal allocation concealment procedures were not implemented. All participants completed an online lecture on the benefits of meditation (*n* = 22) prior to starting the 8-week program.

Adherence was monitored using QR-code attendance logs completed immediately before each meditation session. The QR-code system recorded participant attendance only and did not capture the actual duration of meditation sessions, completion of the full meditation activity, or the quality of engagement during the session.

Feasibility was evaluated descriptively using indicators related to study implementation and participant progression. These indicators included the number of students assessed for eligibility, the number enrolled and randomized, retention through the 8-week intervention, completion of the post-intervention assessment, successful delivery of both meditation modalities, and successful capture of participant attendance through the QR-code system. Because this was an exploratory pilot study, *a priori* numerical thresholds for determining feasibility were not established. Feasibility findings were therefore interpreted descriptively and were intended to inform the design of a future adequately powered trial.

Adherence was operationalized as the number of QR-code-recorded meditation-session attendances completed by each participant during the 8-week intervention. Participants were instructed to complete a minimum of two sessions per week, corresponding to a prescribed minimum of 16 sessions. Adherence was summarized for each intervention group using the total number of recorded sessions, mean number of sessions per participant, standard deviation, range, and the mean number of recorded sessions relative to the prescribed minimum. The QR-code system documented attendance check-in only and did not verify session duration, completion of the full meditation activity, or quality of participant engagement. Accordingly, adherence represents recorded session attendance rather than confirmed completion of the prescribed meditation dose.

Participants were asked to return for post-intervention assessments at the conclusion of the 8-week intervention period, regardless of the number of meditation sessions attended. The post-intervention assessment included administration of the GAD-7 questionnaire and was scheduled for approximately 8 weeks after baseline enrollment for all participants.

### Intervention standardization and fidelity

To enhance consistency across intervention conditions, participants in both groups were instructed to complete guided meditation sessions of approximately equal duration (10 min) and were instructed to complete a minimum of two sessions per week for eight weeks.

Intervention fidelity was promoted through standardized orientation procedures provided to all participants prior to beginning the study. Participants completed an introductory educational session describing the benefits and expectations of meditation practice and were instructed to follow the study-prescribed meditation schedule. Adherence was monitored using QR-code attendance logs completed immediately before each session, allowing session frequency and intervention participation to be tracked throughout the study period.

Meditation sessions were conducted in designated campus meditation rooms intended to minimize environmental distractions. These rooms provided a quiet setting with comfortable seating, subdued lighting, and limited interruptions during meditation sessions.

Meditation content emphasized relaxation, controlled breathing, present-moment awareness, grounding techniques, body awareness, and guided visualization. Participants selected from study-approved meditation playlists developed for the project. The virtual reality (VR) condition delivered this content through immersive 360-degree environments using the Penumbra REAL® i-Series platform, which combined visual and auditory meditation guidance within calming virtual settings. The audio-guided condition utilized comparable mindfulness recordings available through the BurnAlong® platform without immersive visual components. Although individual meditation selections varied based on participant preference, all recordings were selected to reflect similar themes and durations across intervention conditions.

### Statistical analysis

Analyses were organized according to the study objectives. First, feasibility indicators, including screening, enrollment, randomization, retention, completion of post-intervention assessments, intervention delivery, and attendance-data capture, were summarized using frequencies and percentages. Because no *a priori* feasibility thresholds were established, these indicators were interpreted descriptively.

Second, adherence was summarized at both the group and participant levels. Group-level summaries included the total number and percentage of all recorded sessions completed by each intervention group. Participant-level adherence was summarized using the mean, standard deviation, and range of QR-code-recorded session attendances. Mean attendance was also expressed descriptively relative to the prescribed minimum of 16 sessions over the 8-week intervention. No inferential between-group adherence analysis was prespecified; therefore, differences in adherence between the two groups were interpreted descriptively.

Third, preliminary anxiety outcomes were examined. Descriptive statistics, including means and standard deviations, were calculated for baseline and post-intervention GAD-7 scores. Within-group changes from baseline to post-intervention were assessed using paired-samples t tests. Given the small sample size and potential for non-normal distributions, Wilcoxon signed-rank tests were also conducted as sensitivity analyses to evaluate the robustness of the within-group findings. Differences in mean GAD-7 change scores between intervention groups were explored using an independent-samples t test. To directly evaluate differences in anxiety trajectories over time between intervention conditions, a linear mixed-effects model including Condition, Time, and the Condition×Time interaction was conducted, with participant included as a random intercept. Effect sizes and 95% confidence intervals were reported to characterize the magnitude and precision of the observed changes.

Finally, the relationship between adherence and improvement in anxiety was examined using individual participant-level data rather than group-level averages. Adherence was defined as the number of QR-code-recorded session attendances, and improvement was defined as the reduction in GAD-7 score from baseline to post-intervention, with higher positive values representing greater improvement. Because of the small sample and potential for non-normal distributions, a Spearman rank-order correlation was used. All statistical tests were two-tailed, and statistical significance was defined *a priori* as *p* < .05. Analyses were conducted using IBM SPSS Statistics.

## Results

### Participant flow and feasibility

A total of 35 students were assessed for eligibility. Thirteen students were excluded: 10 did not meet the eligibility criteria or were otherwise ineligible because of medical conditions, and three declined participation. Twenty-two students were enrolled and randomly assigned to the VR-guided meditation group (*n* = 11) or the audio-guided meditation group (*n* = 11). All 22 randomized participants completed the 8-week study and post-intervention assessment, corresponding to 100% retention among randomized participants. (Table 1)

**Table 1:** Feasibility and participant-progression indicators.

Feasibility indicator	Result
Students assessed for eligibility	35
Students excluded	13
Students enrolled and randomized	22
VR-guided meditation group	11
Audio-guided meditation group	11
Participants completing the post-intervention assessment	22
Retention among randomized participants	100%
Recorded meditation-session attendances	226
Intervention modalities successfully delivered	2 of 2

Feasibility indicators were evaluated descriptively. No *a priori* numerical feasibility thresholds were established. Recorded meditation-session attendance was obtained from QR-code check-in logs and did not verify session duration or full completion.

Both intervention formats were delivered as planned using the designated campus meditation rooms, Penumbra REAL® i-Series headsets for the VR condition, and the BurnAlong® platform for the audio-guided condition. QR-code attendance records were obtained during the intervention period, resulting in 226 recorded meditation-session attendances. These findings support the practical implementation of both intervention modalities within the institutional setting. However, because *a priori* feasibility thresholds were not established, the findings are interpreted descriptively rather than as evidence that predetermined feasibility criteria were met.

### Participant characteristics

The 22 participants had a mean age of 24.0 years. Eighteen participants were female (81.8%), and four were male (18.2%). Baseline GAD-7 scores were descriptively similar between groups, with a mean of 12.00 (SD = 2.32) in the VR-guided meditation group and 11.91 (SD = 2.55) in the audio-guided meditation group (Table 2).

**Table 2:** Baseline demographic and clinical characteristics of participants.

Variable	VR med (*n* = 11)	Audio med (*n* = 11)	Total (*N* = 22)
Age, years, mean	23.5	24.5	24
Female, *n* (%)	9 (81.8%)	9 (81.8%)	18 (81.8%)
Male, *n* (%)	2 (18.2%)	2 (18.2%)	4 (18.2%)
Baseline			
GAD-7 mean ± SD	12.00 ± 2.32	11.91 ± 2.55	11.95 ± 2.40

Values are presented as mean ± standard deviation (SD) or number (percentage). GAD-7 = Generalized Anxiety Disorder-7 questionnaire. Percentages may not total exactly 100% due to rounding. The overall baseline GAD-7 mean and SD were calculated from the combined study sample.

### Meditation-session adherence

Although participants were instructed to complete a minimum of 16 meditation sessions during the intervention period, adherence varied substantially. Attendance ranged from 1 to 17 sessions in the VR-guided meditation group and from 1 to 18 sessions in the audio-guided meditation group. These findings indicate that many participants did not complete the prescribed number of sessions. However, all 22 randomized participants completed the post-intervention assessment and provided complete GAD-7 data at baseline and at the 8-week follow-up. Consequently, no outcome data were missing for the primary anxiety measure, although adherence to the meditation intervention varied substantially among participants.

Participants completed a total of 226 recorded meditation session attendances during the 8-week intervention. The VR-guided meditation group accounted for 132 recorded sessions, representing 58.4% of all recorded sessions, whereas the audio-guided meditation group accounted for 94 recorded sessions, representing 41.6% of all recorded sessions (Table 3).

**Table 3:** Aggregate recorded meditation-session attendance by intervention group.

Group	Total recorded sessions	Percentage of total sessions
VR meditation	132	58.4%
Audio meditation	94	41.6%
Total	226	100%

Values represent the total number of recorded meditation-session attendances during the 8-week intervention period. Percentages indicate the proportion of all recorded sessions completed by each intervention group.

At the participant level, the VR-guided meditation group completed a mean of 12.00 recorded sessions per participant (SD = 5.42; range, 1–17), whereas the audio-guided meditation group completed a mean of 8.55 sessions per participant (SD = 5.22; range, 1–18). (Table 4) Relative to the prescribed minimum of 16 sessions, these means corresponded to 75.0% of the prescribed minimum in the VR group and 53.4% in the audio-guided group. Across both groups, 226 sessions were recorded out of 352 prescribed minimum sessions, corresponding to 64.2% of the prescribed minimum at the aggregate level.

**Table 4:** Meditation session adherence by intervention condition.

Intervention condition	Participants, n	Prescribed minimum sessions per participant	Mean recorded sessions	Mean percentage of prescribed minimum	SD	Range
VR-guided meditation	11	16	12.00	75.0%	5.42	1–17
Audio-guided meditation	11	16	8.55	53.4%	5.22	1–18

Adherence was operationalized as the number of QR-code-recorded meditation-session attendances during the 8-week intervention. Participants were instructed to complete a minimum of two sessions per week, corresponding to 16 prescribed sessions. Percentages represent the group mean number of recorded attendances divided by the prescribed minimum and do not confirm completion of the full meditation session. The QR-code system recorded check-in attendance only. SD = standard deviation.

Attendance was therefore descriptively greater in the VR-guided meditation group, with a mean difference of 3.45 recorded sessions per participant. However, because an inferential comparison of adherence between groups was not prespecified, this difference should be interpreted descriptively and should not be considered evidence that VR caused greater adherence. In addition, the QR-code system documented check-in attendance only and did not confirm session duration or completion of the full meditation activity.

### Anxiety outcomes

Mean GAD-7 scores decreased from 12.00 (SD = 2.32) at baseline to 6.00 (SD = 3.07) after the intervention in the VR-guided meditation group. In the audio-guided meditation group, mean scores decreased from 11.91 (SD = 2.55) to 5.55 (SD = 2.42) (Table 5).

**Table 5:** Descriptive statistics for GAD-7 scores by intervention group.

Group	n	Pre-Rx mean ± SD	Post-Rx mean ± SD	Mean improvement
VR meditation	11	12.00 ± 2.32	6.00 ± 3.07	6.00
Audio meditation	11	11.91 ± 2.55	5.55 ± 2.42	6.36

GAD-7 = Generalized Anxiety Disorder-7 questionnaire. Pre-Rx = pre-intervention; Post-Rx = post-intervention. Higher GAD-7 scores indicate greater anxiety symptoms. Statistical significance for all analyses was defined as *p* < .05 (two-tailed).

To directly evaluate whether anxiety changed differently across intervention conditions over time, a linear mixed-effects model including Condition, Time, and the Condition×Time interaction was conducted. A significant main effect of Time was observed (z = 6.80, *p* < .001), indicating that GAD-7 scores decreased significantly from baseline to post-intervention across both intervention groups. The Condition×Time interaction was not statistically significant (z = −0.28, *p* = .783), suggesting that reductions in anxiety did not differ significantly between the VR-guided meditation and audio-guided meditation conditions (Table 6). The absence of a significant Condition×Time interaction was consistent with the nonsignificant between-group comparison of change scores reported below.

**Table 6:** Linear mixed-effects model examining condition, time, and the condition×time interaction for GAD-7 scores.

Effect	Estimate, *β*	Standard error	Test statistic, z	*p* Value	95% CI
Time	6.36	0.94	6.80	<.001	4.53 to 8.20
Condition	0.46	1.11	0.41	.682	−1.72 to 2.63
Condition×Time	−0.36	1.32	−0.28	.783	−2.96 to 2.23

GAD-7 = Generalized Anxiety Disorder-7 questionnaire; CI = confidence interval. The model included condition, time, and the Condition×Time interaction as fixed effects, with participant included as a random intercept. The audio-guided condition and post-intervention assessment served as the reference categories. The significant main effect of time indicates that GAD-7 scores decreased from pre-intervention to post-intervention across conditions. The nonsignificant Condition×Time interaction indicates that the magnitude of change did not differ significantly between the VR-guided and audio-guided meditation groups. Statistical significance was defined as *p* < .05, two-tailed.

### Within-group comparisons

Both intervention groups demonstrated significant reductions in anxiety following the 8-week intervention. Participants in the VR meditation group demonstrated a significant reduction in GAD-7 scores (*t*(10) = 6.87, *p* < .001). The Wilcoxon signed-rank test confirmed this finding (*p* = .001). Similarly, participants in the audio guided meditation group demonstrated a significant reduction in anxiety (*t*(10) = 6.41, *p* < .001), which was also confirmed by the Wilcoxon signed-rank test (*p* = .001). (Table 7)

**Table 7:** Within-group comparisons of GAD-7 scores.

Group	Mean reduction	Paired t-test	*p*-value	Wilcoxon *p*-value	Interpretation
VR meditation	6.00	*t*(10) = 6.87	<.001	.001	Significant reduction in anxiety
Audio meditation	6.36	*t*(10) = 6.41	<.001	.001	Significant reduction in anxiety

GAD-7 = Generalized Anxiety Disorder-7 questionnaire. Statistical significance was defined as *p* < .05 (two-tailed). Both intervention groups demonstrated statistically significant reductions in anxiety following the intervention.

### Between-group comparison

An independent-samples t-test was used to examine the level of improvement in anxiety between the VR meditation and audio guided meditation groups. There were no significant differences in change scores between groups (t(20) = 0.27, *p* = .786), however, given the pilot nature of the study and limited sample size, this finding should not be interpreted as evidence of equivalence between interventions. (Table 8)

**Table 8:** Comparison of change in GAD-7 scores between intervention groups.

Comparison	Test	Test statistic	*p*- value	Interpretation
VR vs. Audio Meditation	Independent *t*-test	*t*(20) = 0.27	786	No significant difference between groups

Statistical significance was defined as p < .05 (two-tailed). No statistically significant difference in GAD-7 change scores was detected between intervention groups.

The exploratory Spearman correlation did not identify a statistically significant association between the number of recorded meditation-session attendances and improvement in GAD-7 scores, *ρ* = −.195, *p* = .385 (Table 9). Thus, within this small pilot sample, participants with a greater number of recorded attendances did not necessarily demonstrate greater reductions in anxiety symptoms. This analysis was based on individual participant-level data rather than group averages.

**Table 9:** Correlation between meditation frequency and improvement in GAD-7 scores.

Analysis	Correlation coefficient	*p*-value	Interpretation
Spearman Correlation	*ρ* = −0.195	.385	No significant relationship

GAD-7 = Generalized Anxiety Disorder-7 questionnaire. Improvement was defined as the reduction in GAD-7 score from baseline to post-intervention, with higher values indicating greater reductions in anxiety symptoms. Statistical significance was defined as *p* < .05 (two-tailed).

Both intervention groups showed significant reduction of anxiety symptoms after the 8-week meditation intervention. The effect size for the VR meditation group was very large (Cohen's d_z_ = 2.07) and the effect size for the audio guided meditation group was very large (Cohen's d_z_ = 1.93), indicating that both interventions resulted in significant improvements in GAD-7 scores. In contrast, the between-group effect size was negligible (Cohen's d = 0.12), suggesting no statistically significant advantage for either intervention over the other. The mean reduction in GAD-7 score was 6.00 points (95% CI, 4.05 to 7.95) in the VR group and 6.36 points (95% CI, 4.15 to 8.57) in the audio-guided meditation group. The confidence intervals were similar in magnitude and consistent with no statistically significant between-group difference. Taken together, these findings indicate that anxiety symptoms decreased over the study period in both intervention groups. (Tables 10, 11)

**Table 10:** Effect sizes for anxiety outcomes.

Comparison	Cohen's *d*	Magnitude
VR meditation (Pre vs. Post)	2.07	Very large
Audio meditation (Pre vs. Post)	1.93	Very large
VR vs. Audio Meditation (Change Scores)	0.12	Trivial

Cohen's d was calculated to estimate the magnitude of observed changes in anxiety outcomes. Within-group effect sizes represent pre- to post-intervention changes in GAD-7 scores for each intervention condition. The between-group effect size represents the difference in GAD-7 change scores between the VR-guided meditation and audio-guided meditation groups. Conventional benchmarks for interpretation were used: 0.20 = small, 0.50 = medium, and 0.80 = large effect size.

**Table 11:** Mean change in GAD-7 scores with 95% confidence intervals.

Group	Mean change	95% Confidence interval
VR meditation	6.00	4.05 to 7.95
Audio meditation	6.36	4.15 to 8.57

Mean change represents the reduction in GAD-7 score from baseline to post-intervention. Positive values indicate improvement (reduced anxiety). Confidence intervals were calculated using paired-sample statistics with *n* = 11 participants per group.

## Discussion

The first objective of this pilot study was to evaluate the feasibility of implementing VR-guided and audio-guided meditation interventions within an academic health sciences institution. Thirty-five students were assessed for eligibility, 22 were enrolled and randomized, and all 22 completed the 8-week intervention and post-intervention assessment. Both intervention modalities were delivered within the designated meditation rooms, and the QR-code system captured 226 session-attendance records. Collectively, these findings demonstrate that the study procedures and both intervention formats could be implemented in this institutional setting. However, because feasibility benchmarks were not established *a priori*, the findings should be regarded as descriptive indicators of implementation rather than evidence that formal feasibility criteria were met.

The second objective was to describe adherence to the two meditation modalities. Adherence was operationalized as QR-code-recorded meditation-session attendance. Participants assigned to VR-guided meditation completed a mean of 12.00 recorded sessions, equivalent to 75.0% of the prescribed minimum of 16 sessions. Participants assigned to audio-guided meditation completed a mean of 8.55 sessions, equivalent to 53.4% of the prescribed minimum. Across both groups, recorded attendance represented 64.2% of the prescribed minimum number of sessions.

The VR-guided group demonstrated descriptively greater attendance, completing an average of 3.45 more recorded sessions per participant than the audio-guided group. This finding may indicate that the immersive format was acceptable or appealing to some participants, but it does not establish that VR increased adherence because the difference was not evaluated using a prespecified inferential comparison. In addition, attendance varied substantially within both groups, and the QR-code system recorded check-in only. It did not verify the duration of meditation, completion of the selected activity, or quality of engagement. Consequently, the adherence findings describe participation frequency rather than the full meditation dose received.

An important finding of this pilot study was the variability in adherence. Although participants were instructed to complete a minimum of two meditation sessions per week for eight weeks, actual attendance varied widely, with some participants recording only a single session during the intervention period. These findings suggest that while implementation of both meditation modalities was feasible, adherence to the prescribed intervention schedule was more limited than anticipated. Consequently, the study should be interpreted as evaluating access to and engagement with guided meditation rather than full completion of the intended meditation dose among all participants.

The third objective was to estimate preliminary changes in anxiety symptoms. GAD-7 scores decreased over the 8-week period in both intervention groups. The mean reduction was 6.00 points in the VR-guided meditation group and 6.36 points in the audio-guided meditation group. Within-group tests were statistically significant, and large standardized within-group changes were observed. However, the study did not include an inactive or treatment-as-usual control group. The observed changes therefore cannot be attributed definitively to either meditation intervention, as regression to the mean, natural variation in anxiety, concurrent support, or other unmeasured influences may have contributed.

The exploratory between-group comparison did not detect a statistically significant difference in change scores, t(20) = 0.27, *p* = .786, Cohen's d = 0.12. Consistent with this finding, the linear mixed-effects model demonstrated a significant main effect of time but no significant Condition×Time interaction, indicating that reductions in anxiety over time did not differ significantly between intervention modalities. Because this pilot study was not powered to detect small-to-moderate differences between two active interventions, the absence of statistical significance should not be interpreted as evidence that the modalities are equivalent. Rather, the estimate provides preliminary information for planning a larger trial.

Our findings are consistent with prior research demonstrating that meditation can reduce stress and anxiety among college and medical students (7, 10). They also correspond to research that shows that immersive VR environments help relaxation by reducing external distractions and improving concentration (14, 19). VR has been proposed to enhance meditation through increased immersion and presence; however, these advantages did not result in larger reductions of generalized anxiety compared to traditional audio guided meditation.

There are a few reasons that could help explain why the two therapies had similar outcomes. Both groups of participants received identical introductory meditation instruction and were prescribed meditation sessions of similar duration and frequency. Because both groups received guided meditation interventions of similar duration and frequency, the study compared two active intervention modalities. Therefore, the findings provide information regarding differences between delivery formats rather than the independent effect of meditation itself.

The fourth objective was to explore whether meditation-session attendance was associated with improvement in anxiety. The number of QR-code-recorded attendances was not significantly associated with improvement in GAD-7 scores. Therefore, the descriptively greater attendance observed in the VR group did not correspond to evidence that participants who attended more sessions experienced greater anxiety reduction.

Several explanations are possible. The number of recorded sessions may not adequately represent meditation exposure because attendance logs did not document session duration, completion, or engagement quality. Anxiety outcomes may also have been influenced by baseline symptom severity, prior meditation experience, coping strategies, outside meditation practice, or other concurrent supports. Given the small sample, this exploratory correlation should be interpreted cautiously.

Within group effect sizes were quite high for both intervention groups. The Cohen's d was 2.07 for the VR group and 1.93 for the audio led meditation group demonstrating significant reductions in anxiety in both groups. Conversely, the between-group effect size was small (Cohen's d = 0.12). Given the pilot sample size, this estimate should be interpreted cautiously and does not establish equivalence between intervention modalities.

Although VR-guided meditation did not produce greater anxiety reduction than audio-guided meditation, both interventions appeared feasible to implement within the institutional wellness setting. Participants assigned to the VR condition recorded more session attendances on average. This descriptive pattern warrants further investigation using formal measures of acceptability, usability, motivation, and intervention engagement. Students who struggle to concentrate in traditional meditation settings may find VR to be a more interesting experience that reduces distractions. At the same time, audio guided meditation remains an attractive choice as it is inexpensive, widely available and does not require specific equipment. This provides institutions with flexibility to offer either strategy depending on resources and student preference. Importantly, neither modality demonstrated a statistically significant advantage over the other with respect to anxiety outcomes in this pilot sample.

The findings also highlight LSU Health Shreveport's continuing investment in preventive mental health efforts. The Office of Institutional Wellness and specialized meditation rooms offer students and trainees accessible opportunity to participate in stress management and self-care. Providing a range of evidence-based meditation practices allows universities to serve a heterogeneous student body, while increasing resilience and psychological well-being.

Several limitations should be considered when interpreting these findings. First, the study involved a relatively small sample and was not powered to detect modest differences between two active intervention conditions. Consequently, the absence of significant between-group differences should not be interpreted as evidence of equivalence. Rather, the findings should be viewed as preliminary and hypothesis-generating. The primary contribution of this pilot study is the demonstration of feasibility and participant adherence to guided meditation interventions delivered through different modalities.

Recruitment nevertheless presented an important implementation challenge. Thirteen of the 35 students assessed did not proceed to randomization, including three who declined participation. Informal feedback suggested that anxiety, concerns about participating in mental health research, stigma, time constraints, or participation burden may have influenced some students’ willingness to enroll. These observations are consistent with prior research demonstrating that college students often face barriers to seeking mental health services, including concerns about stigma, a preference to manage problems independently, and reluctance to engage in formal mental health interventions (17) Because these factors were not systematically measured, they should be considered observations that can inform future recruitment strategies rather than formal study findings.

Future studies should utilize larger powered samples and include recruitment strategies designed to address potential barriers to participation. Understanding factors that influence enrollment and retention may be particularly important when evaluating mental health interventions among student populations.

Further, the participants were recruited from one academic health center and hence the findings may not be generalizable to other institutions or student groups. Also, the intervention was limited to 8 weeks in duration and so could not be used to assess long-term adherence and sustainability of anxiety reduction. In addition, attendance was the only measure of adherence. The QR-code system captured session check-in attendance but did not verify the duration of meditation practice, completion of the entire meditation session, or the quality of participant engagement. As a result, the adherence measure reflects participation frequency rather than the total amount or quality of meditation practice.

In addition, because outcome assessments were completed regardless of intervention adherence, anxiety outcomes reflect an intention-to-follow-up sample rather than only participants who completed the prescribed meditation dose.

Finally, measures of participant satisfaction, perceived usability, acceptability, and user experience were not collected and therefore could not be evaluated. Future studies should include these outcomes to better assess implementation potential.

## Conclusion

This pilot study examined the feasibility, adherence, and preliminary anxiety outcomes associated with virtual reality (VR) guided meditation and audio-guided meditation among medical, graduate, and health professions students at LSU Health Shreveport. Participants in both intervention groups demonstrated substantial reductions in GAD-7 scores over the 8-week study period; however, because no inactive or treatment-as-usual control group was included, these findings cannot establish that the interventions alone were responsible for the observed improvements.

No statistically significant differences were detected between the VR and audio-guided meditation groups. However, the study was not powered to identify small-to-moderate between-group differences, and therefore the absence of significant findings should not be interpreted as evidence of equivalence between intervention modalities. Participants assigned to VR meditation completed more recorded sessions on average than participants assigned to audio-guided meditation; however, this descriptive difference should be interpreted cautiously because adherence comparisons were not evaluated inferentially. Session attendance was not significantly associated with anxiety outcomes, indicating that greater adherence alone did not explain changes in GAD-7 scores.

The primary contribution of this pilot study lies in its assessment of feasibility and adherence to guided meditation interventions among health professions students. Recruitment challenges highlighted an important consideration for future research. Informal feedback from students suggested that anxiety about participating in a study focused on anxiety and mental health may have discouraged enrollment among some otherwise eligible participants. Although these observations were anecdotal and not systematically assessed, they suggest that concerns related to mental health stigma, participation burden, or anxiety itself may influence recruitment in studies involving student well-being.

Overall, the findings suggest that both VR and audio-guided meditation are feasible approaches for delivering mindfulness-based interventions in graduate and professional student populations. Future adequately powered randomized controlled trials that include a non-intervention comparison group, formal measures of acceptability and usability, and longer follow-up periods are needed to determine intervention efficacy, identify potential differences between delivery modalities, evaluate long-term adherence and outcomes, and examine broader dimensions of student well-being, including stress, resilience, burnout, sleep quality, and academic performance.

## Data Availability

The raw data supporting the conclusions of this article will be made available by the authors, without undue reservation.
